# Coronial Practice, Indigeneity and Suicide

**DOI:** 10.3390/ijerph15040765

**Published:** 2018-04-16

**Authors:** Gordon Tait, Belinda Carpenter, Stephanie Jowett

**Affiliations:** 1 Faculty of Education, School of Cultural and Professional Learning, Queensland University of Technology, Brisbane, QLD 4001, Australia; g.tait@qut.edu.au; 2 Faculty of Law, Queensland University of Technology, Brisbane, QLD 4001, Australia; 3 Australian Centre for Health Law Research, Faculty of Law, Queensland University of Technology, Brisbane, QLD 4001, Australia; s.jowett@qut.edu.au

**Keywords:** suicide, indigenous suicide, aboriginal Australian, youth suicide, mental health, coroner, legal decision making

## Abstract

All available data suggest that, like many other Indigenous peoples, Australian Aborigines are significantly more likely to kill themselves than are non-Aboriginal Australians. This statistical disparity is normally positioned an objective, ontological and undeniable social fact, a fact best explained as a function of endemic community disadvantage and disenfranchisement. This research explores the possibility that higher-than-normal Aboriginal suicide rates may also be a function of coronial decision-making practices. Based upon in-depth interviews with 32 coroners from across Australia, the following conclusions emerged from the data. First, coroners have differing perceptions of Indigenous capacity, and are less likely to have concerns about intent when the suicide is committed by an Indigenous person. Second, coroners have identified divergent scripts of Indigenous suicide, particularly its spontaneity and public location, and this supports rather than challenges, a finding of suicide. Third, the coronial perception of Indigenous life is a factor which influences a suicide determination for Indigenous deaths. Finally, the low level of Indigenous engagement with the coronial system, and the unlikelihood of a challenge to the finding of suicide by Indigenous families, means that a coronial determination of suicide is more likely.

## 1. Introduction

It is not in dispute that Indigenous Australians have been—and continue to be—subject to significant social, cultural, political, legal, medical and psychological trauma [1]. Through the effects of introduced disease, military resistance, displacement, and extra-judicial killings, in the 230 years since colonisation began, the Indigenous population has fallen from an estimated 750,000 to a low point of 120,000 at the beginning of the 20th century, before reaching the current levels of approximately 786,689 [2]. At the present time, Indigenous infant mortality rates are five times those for non-Indigenous Australians (4.2% vs. 0.8%); the unemployment rate for Indigenous Australians is 4.2 times higher than non-indigenous Australians; their life expectancy is also over ten years less [3]. Indigenous Australians are 13 times more likely to be imprisoned [4]. Of the estimated 250 Indigenous languages which existed in the late 18th century, only 13 remain in daily use [5].

Perhaps unsurprisingly, this statistical portrait of a comprehensive Indigenous crisis extends to the issue of suicide. In Australia, Indigenous suicide rates are currently twice that of the non-Indigenous [6]. This is of particular importance as suicide rates are frequently directly correlated to the health of the social body. According to this logic, struggling, traumatised communities are likely to have high suicide rates, and high suicide rates are likely to be deemed indicative of struggling, traumatised communities [7]. While this relationship constitutes an important research topic in its own right, this paper does not seek to address the validity of the logic behind this correlation, rather it examines the veracity of the data upon which particular examples of this correlation are often founded.

The specific focus here on Indigenous suicide has emerged from a broader project questioning the status of suicide rates as ‘objective measures’ of the health of given cohorts within the social body —and as such, this research therefore takes its place within the social constructionist literature on the category of suicide [8,9,10]. Suicide rates have long been heavily questioned by almost all those involved in the field, who point to ongoing systemic underestimations of anywhere between 15% to 50%—making such rates “not only unreliable but useless for the purpose to which they are put in sociological research” [11]. In Australia, this dissatisfaction with official suicide rates has even resulted in the establishment of a Senate committee into the issue in 2010, which led to the publication of *The Hidden Toll: Suicide in Australia* [12].

As a result of this, it is suggested here that any unpacking of the complex relationship between social crisis, suicide rates and Indigenous communities, will benefit from a greater understanding of the mechanisms by which those specific suicide rates are produced, and upon which the logic of the argument rests. This is because Coroners’ findings contribute to national suicide statistics in Australia [13]. Indeed, coroners are presently the only legal persons who make routine determinations of suicide so that it may be coded as such [14,15,16]. As a result, the process by which coroners make determinations of suicide is critically linked with policymaking in public health and mental health, as well as planning and funding of suicide prevention strategies [12,17]. To date, most research to investigate the process of suicide determination by coroners has focused on the output of coronial decision-making in the form of secondary analysis of coronial data (see, for example [18]; there is also considerable research in England that follows this approach [19,20]). In contrast, this paper will explore through in depth interviews, how coroners go about making their determinations of suicide—what counts as a suicide, and what does not; what evidence is used to reach that decision; how much weight is placed on different types of evidence; how various domain assumptions may affect the decision-making process—all with specific relation to Indigenous death.

## 2. Methods

This study is informed by interviews with 32 coroners from all states and territories in Australia, except Tasmania. Selective sampling was the basis for coroner involvement in the research. Contact was made with each coronial office through the Chief Coroner who then provided the contact details of coroners willing to participate in the project. As the cohort of sitting coroners is relatively small, past coroners were also interviewed in order to capture a range of experience, expand the sample size and enhance anonymity. Having the support of two State coroners in the initial stages of this research was crucial, and as the list of participants grew, the legitimacy and significance of the research increased. This in turn made it easier to access more coroners to interview. It is often suggested that judicial officers are reluctant to be interviewed, believing that the law rather than the officer is responsible for a legal outcome [21]. This was the case in only one state of Australia, where access to coroners was denied by the Chief Magistrate.

The interviews were semi structured and conducted in the coroners’ offices. Most took one hour to complete. They were recorded and transcribed and then sent back to the coroner for their review. Only one coroner withdrew from the process at this point and their interview was destroyed. Coroners ranged in their professional experience. The longest serving Coroner had been in the job for nearly 20 years, while the shortest serving Coroner had only been employed for six months. Both genders were well represented in the sample with 14 female coroners and 18 male coroners interviewed for the project. Coroners were also located in both rural and urban areas; capturing those adjudging deaths of individuals across a wide range of cultural and socio-economic backgrounds.

The interviews were treated as a social encounter. This means that the interview was understood as producing knowledge rather than simply recording it. The coroners were understood as constructors of knowledge rather than as simple repositories, and the interview itself was a process of meaning making between the coroner and the interviewer. Interviews were thus interpretative, active and collaborative [22]. However, this process was only possible because we followed the rules of “elite interviewing” offered by Mannheim and Rich: use a reflective and conversational tone; plan initial questions carefully; ask questions that could be subject to multiple interpretations; know the subject matter [23].

Knowing the subject matter and having legitimacy is crucial because coroners and other judicial officers are socialised to hide or ignore the decision-making processes that are often of most interest to researchers. Avoiding unproductive interviews thus relies upon the interviewer establishing credibility and the coroner accepting the interviewer’s status as “quasi insider” [24]. This was achieved by our academic status, our previous published research and external grants on the topic, and our knowledge of the workings of the system.

In interviewing high-status individuals, who were willing to be very open in discussing a sensitive subject, anonymity was a serious consideration in this study. To protect the identity of the interviewees, the numbers assigned to participants were rotated across publications to avoid cross-referencing of quotes. Where not required to illustrate a particular point, the location and background of the coroners was deliberately omitted.

The interview questions centred upon conceptualisations of suicide, data coding and entry procedures, the processes and problems of suicide adjudgment, alternative ways of managing Coronial approaches to suicide determination, and the specific problems association with regards high-risk groups, such as Indigenous Australians. Interviews continued until saturation was reached. In addition, the authors conducted an analysis of the documents that organise Coronial practices and responsibilities, as they relate to reaching a finding of suicide: state legislation, policy documents, professional guidelines, training materials, and data coding and entry procedures [25].

Thematic analysis was the key process utilised in this research and an inductive approach to the data was favoured [26]. Thematic analysis of the transcripts began with a process of schematic coding, which required all transcripts to be read in their entirety by the research team. Themes were identified through a series of discussions between the research team where both dominant and emergent themes were identified and then reviewed. It became clear during this process that coroners used information differently when suicide determination of Indigenous Australians was the focus. Moreover, some coroners had much less experience than others with Indigenous suicide determination and this was often associated with the location of the coroner, both in terms of the state/territory and/or whether they were working in a rural or urban space. Dominant themes identified in the context of the coronial determination of Indigenous peoples and suicide included the following: differing perceptions of capacity, particularly when children and alcohol were considered; divergent scripts of suicide for Indigenous Australians; coronial perceptions of social disadvantage and their role in the likelihood of a suicide occurring; and the low level of engagement by Indigenous families with the system. Each of these were identified as leading to differing outcomes when Indigenous and non-Indigenous Australians were discussed.

## 3. Results/Discussion

The results are divided into two main sections. The first covers some general findings about the practical coronial determination of suicide, including how suicide is conceptualised within coronial practice, and from there, how a finding of suicide is actually adjudged (and thence how suicide is statistically counted). From there, specific issues relating to the determination of indigenous suicide will be discussed. In both cases, quotes from the coroners themselves will be used to illustrate the central findings.

### 3.1. General Findings

#### 3.1.1. There Is an Ongoing Reluctance to Reach a Finding of Suicide

Coroners appear to avoid the finding of suicide whenever possible, or whenever another finding is plausible. They are often under varying degrees of pressure from families—particularly at inquest—to reach open findings, or findings of accident, as alternatives [27]. Also, coroners are aware that there remains significant stigma against those who die by suicide, and by proxy, their families, a situation they find themselves trying to avoid [28]. As a consequence, for the most part, coroners are perfectly aware that official suicide rates constitute a significant underestimation.

*“I know I have made decisions which have been intellectually dishonest in order to try and do the kind thing, I guess.”* 

(Coroner 1)

*“No to be totally honest, I think I’ve ducked and weaved and put it down to an unfortunate accident, even though I thought it was a suicide.”* 

(Coroner 7)

*“Probably out of being humanly weak, I would probably take into account what I understand to be the circumstances of the family.”* 

(Coroner 18)

Coroners are, of course, not writing fiction and do make suicide findings, often and regularly. Part of the problem for coroners is balancing the competing demands of accurate manner of death findings with the need for care and sensitivity toward the family. In suicide verdicts, these are made more complicated by the difficulty of determining intent in the context of a highly stigmatised death and the various and varying definitions of suicide that currently exist [29]. There is thus significant ambiguity or “wriggle room” in a finding of suicide, ambiguity that is also acknowledged in the literature [30].

#### 3.1.2. There Are Significant Issues Relating to Judicial Consistency

Coronial decision-making is contingent upon a wide range of factors, such as the individual coroner’s experience, gender, state or territory where they practice, degree of training, constituency, personal background and personality. This has resulted in a degree of variance between coroners as to what kind of evidence would be required in order to satisfy themselves that a specific death was in fact a suicide. Two issues are particularly evident:

##### Standard of Proof

In Australia, the standard of proof required to reach a finding of suicide is the civil standard of “on the balance of probabilities” [25]. However, precisely what this means varies considerably between individual coroners. The application of a standard of proof in suicidal determination is inextricably bound up with the historical consequences of a finding of suicide, where until recent decades its criminalisation and impacts on life insurance policies and religious burial rites meant that the application of the *Briginshaw Principle* was relevant [25].

*“I’m always a bit staggered when people tell me how hard it is to do beyond a reasonable doubt, because I don’t think that’s hard at all. What’s really quite hard is balance of probabilities …. That can lead to some real difficulties in coroners matters around suicide and lack of understanding of that test.”* 

(Coroner 6)

*“So if I say I find that its suicide, the person intended to take their own life, what I mean is I’m satisfied to that Briginshaw standard that that’s what’s going on. If I don’t say that then that means I’m not satisfied, that doesn’t mean it’s not a suicide.”* 

(Coroner 31)

Much of the concern from coroners about the standard of proof, however, comes not so much from its history of criminalisation or the religious or financial concerns but from the stigma for the families left behind. Research supports the idea that the finding of suicide is not morally neutral for many families and this has been found to impact on the suicide determination of coroners [31].

*“The only issue of stigma I feel is whether they feel stigmatized by it. That makes me think hard before I make a decision about whether it’s a suicide or not.”* 

(Coroner 22)

*“The blame the family feels and the crazy presumption that if you were a caring loving family you would have foreseen this and stopped it. There’s the social stigma there, a huge one.”* 

(Coroner 21)

##### Definitions of Suicide

There is no standard definition of suicide deployed across all Australian jurisdictions [25]. This is perhaps not surprising given the range of definitions in use across the literature [29]. The definitions used in the Australian coronial context appear to have been taken from a variety of sources—civil law, dictionaries, ‘common sense’, academic texts, internal memos and bench-books [25]. As a consequence, whether or not a death is adjudged as a suicide, is often contingent simply upon which definition is being used. The issue of intent provides a good example here, where coroners offer divergent views on its requirement for the determination of suicide.

*“I don’t think you actually require an intent to kill yourself for suicide. All you have to intend is a dangerous act against yourself and the consequence is death. What your intent or what your motivation was is beside the point.”* 

(Coroner 16)

*“There has to be evidence that the person intended to die, or they intended to take their life.”* 

(Coroner 14)

Different coroners thus appear to apply the notion of intent in very different ways. There are, of course, many circumstances where determining the intent of a person at the time that they commit a supposed suicidal act may be inherently difficult based on the nature of the act (overdoses, drownings, single vehicle accidents). Also, it is important to note that suicidal intention itself—as distinct from its determination—is far from being a clear cut concept, given that “multiple and contradictory intentions can coexist” [32]. Mental illness, however, would not normally deprive a person of their ability to appreciate the consequences of their actions, with depression a known risk factor for suicide. According to Cavanagh et al. (2003) between 47% and 75% of suicides examined in their study were attributable to a mental disorder, normally depression [33]. However, the particular case of psychosis is generally deemed to preclude a finding of suicide for coroners, based on the difficulty of identifying the requisite intent.

*“If you’re having a psychotic episode and you think you’re superman, and you’re going to stop the train … that’s not suicide.”* 

(Coroner 20)

*“So a psychotic episode, I’m jumping off a building, I think I’m a bird. Am I committing suicide?” (Interviewer) “Yes.”* 

(Coroner 3)

However, as the quotes above demonstrate, this is not necessarily an agreed upon principle for coroners, and also is not well supported by the literature, with a range of studies identifying suicide as the main cause of premature death among individuals experiencing psychosis [34].

#### 3.1.3. Family Engagement with the Process

Since the mid-1980s, as Australian coronial Acts have been updated, families have been invited into the process in varying ways [35]. Motivated by coronial legislation which stresses the rights of family members to be involved in decisions concerning the deceased, it is increasingly being suggested that a coroner’s work is intimately connected with well-being and the emotional closure of the family [36]. Certainly, in the context of suicide determinations, research has demonstrated that coroners are impacted upon by familial resistance to a finding of suicide [37]. Moreover, this resistance can be forceful and unrelenting.

*“The wife from the outset had been vehemently opposed to suicide, knowing that it was quite a strong possibility, and the constant contact with the counsellors indicating that she would probably commit suicide if there was a suicide finding on it—which obviously put some pressure on me to make that decision … in the end I found open.”* 

(Coroner 32)

*“I can say this: there is lot of family pressure, and they will write to you, and it can be very hard to stay strong and say no.”* 

(Coroner 6)

This sense of responsibility for the well-being of the family of the deceased leads to coroners enacting a form of “therapeutic jurisprudence”. In such a scenario, the law is understood not merely as a set of codes to be followed without reflection. Rather legal institutions, and judicial officers are deemed to have responsibility for the mental and emotional well-being of participants in the process [37]. According to this approach, it would be inappropriate for a coroner to reach a finding of suicide without considering the impact of this finding on those close to the deceased. This means that despite the formal legal function of their coronial determinations, coroners can also function as an informal therapeutic filter through which the factual circumstances of a death are directed, in order to try and manage the individual emotional impact.

#### 3.1.4. Ontological and Epistemological Problems with the Notion of ‘Suicide’

A number of coroners argued that the binary of suicide/not suicide is insufficient for adequately covering the range of behaviours encompassed by the notion of suicide. In particular, a third possible category representing “self-manslaughter”—behaviour so dangerous that death was a likely outcome, irrespective of actual intent—is widely regarded as a useful proposed addition. Other coroners regard the category of suicide itself as inherently flawed, in that it covers such a wide range of potential behaviours, outcomes and intentions, that a single term cannot hope to represent them all.

*“I just don’t like the fact that there’s such a small number of categories. It would be nice to have more, but … I don’t know what those more would be.”* 

(Coroner 21)

*“There’s no value at all in saying, suicide, not suicide. It’s rubbish. The only people that are interested in that are the ABS and the people that go around saying—the people that make decisions about how you expend public money on the basis of how many suicides there are.”* 

(Coroner 17)

Suicide verdicts thus depend on a range of relevant cues from the biography of the deceased to the content of the death. The capacity of a coroner to determine the death as accidental or undetermined, rather than as a suicide, is made possible due to the varying definitions of suicide which exist and the difficulty of determining intent in the context of risk taking behaviour, for example. Add vocal family resistance to this and it is apparent that determining a suicide is far from a simple task. This is made more difficult when cultural difference in the form of Indigeneity is added to this complex process.

### 3.2. Specific Findings Relating to Indigeneity

Within a general framework of issues relating to the coronial determination of suicide, there exist some specific observations about Indigenous suicide, observations which speak directly to the accuracy of Indigenous suicide statistics, and to the differential ways that suicide is determined on the basis of racial and ethnic identity. Five issues will be addressed here: the different scripts of suicide between Indigenous and non-Indigenous Australians; the different role Indigenous families play in the coronial system; the role of social disadvantage in Indigenous Australians on suicide findings; the difficulty of utilising the balance of probabilities in a consistent manner; and, the different ways in which capacity may be interpreted by coroners when the deceased is Indigenous.

However, there are a number of points to make about these findings before proceeding to their discussion. First, they are based on the perception of the coroners themselves as opposed to any “truth” about Indigenous suicide. In fact, this is precisely the point of the research—that the category of suicide and thus its coronial determination is socially produced rather than existing as an objective reality. Coronial perceptions are integral to their determinations.

Second, Indigenous people are over-represented in the coronial systems of Australia and this is due in large part to structural factors such as endemic violence, poor access to health care, low life expectancies and high rates of chronic disease [38]. Moreover, in Australia, it is police who are legislatively required to investigate all coronial deaths, but this occurs within a long and well documented history of poor relations between police and Indigenous people [39]. As a consequence, identification and negotiation may be particularly onerous for Indigenous families and may mean that coroners rely on stereotypical understandings—a situation recognised by at least one coroner during our interviews.

*“But bearing in mind, Indigenous people don’t really tell their story very well. So you’re relying a lot on hearsay and information like that.”* 

(Coroner 14)

Finally, and related to the previous point, many coroners claim to have had little contact with Indigenous suicide, especially those that work in urban areas of the larger Australian cities. This often meant that interviews with these coroners offered very little insight into the elements of an Indigenous suicide.

*“No, look, I haven’t had many cases of Indigenous suicide.”* 

(Coroner 28)

*“I don’t actually think that I’ve had a suicide of a person, Aboriginal or Torres Strait Islander. So, I’m sorry I can’t...”* 

(Coroner 29)

*“We don’t have a very big Indigenous community in [urban area] and I don’t, I wouldn’t have enough to see a trend.”* 

(Coroner 9)

However, the invisibility of Indigenous people in coronial investigations has been noted in previous research and may say as much about the consequences of police investigations and their role in the history of colonisation of Indigenous people, as any reality of the cultural nature of the death [40].

#### 3.2.1. Scripts of Indigenous Suicide

According to the coroners interviewed, the usual scripts of suicide do not appear to apply to Indigenous Australians to the same degree as non-Indigenous. Whereas the normal expectations around suicide include such factors as depression, suicide notes and previous attempts, these factors appear far less relevant for Indigenous Australians. Previous Australian research does support this understanding, where suicide notes and diagnosed mental illness, for example, were found to not be useful markers of Indigenous suicide [41]. Coroners do seem aware of this, especially those who are in states with higher Indigenous populations.

*“I wouldn’t dream of looking for a suicide note in an Aboriginal person, that would be just craziness because they never do that so why would you look for that.”* 

(Coroner 10)

*“But interestingly, you’re not going to find an aboriginal suicide where they’re—well, some of them, I’m generalizing—but most of them are not on anti-depressants and they don’t have a history of mental illness and they haven’t been treated generally.”* 

(Coroner 25)

It has been recognised that aboriginal suicide behaviours, especially in remote Australia, can present as very different phenomena to suicidal behaviours in mainstream Australian society [42]. Suicide often appears as a way of expressing retaliation, rather than an immediate wish to die. Similarly, threats of suicide, often over seemingly trivial matters, are often acted upon in an impulsive manner. Finally, given that the lethal method most often used by Indigenous people is hanging, these acts often end in death. Coroners interviewed for this research were also aware of these issues, but this did not unsettle their finding of intent.

##### Suicides Are Often over Trivial Matters, and Spontaneous

Coroners have suggested that Indigenous suicides may be more easily triggered than non-Indigenous suicide. They can occur after minor disagreements, arguments or disappointments. They are not necessarily accompanied by previous self harm, and are often entirely unanticipated by their family and community. Furthermore, they often appear to be a spur of the moment action. For example, Hunter et al. (2006) suggests that a “typical” Indigenous case includes some sort of interpersonal conflict with members of the family or with a partner, which to an outsider might seem “trifling” [43].

*“The other thing I noticed was that they required far less of a trigger for suicide than perhaps the European community.”* 

(Coroner 18)

*“It’s becoming the norm and it is—it’s triggered. It’s very easy. People flip that off the tongue. I’m going to kill myself.”* 

(Coroner 24)

*“But one interesting thing is more so than others, it seems to be completely spur of the moment in the sense—I mean, I’m sure there’s a context of a difficult life and a difficult background. But I’ve had files where they say, we were all sitting and drinking and we thought, gee, Jeff has been gone for a while. Then we walked outside and there he was hanging off the back clothesline.”* 

(Coroner 25)

##### Suicides May Be Targeted

Indigenous suicides are often relational, in that they speak less about an internal mental state, and more about anger within a given relationship. A person who suicides may be only one side of the event, with the other side being constituted by the intended target of the death. In his seminal text on Indigenous suicide, Tatz (2005:75) calls such suicides “political” in that they are a public declaration of anger or grievance designed to gain a hearing, possibly even a response [39]. Such suicides encompass sentiments such as “you’ll be sorry”, or “you’ll pay for this”.

*“But what has struck me, almost every time … is that they’ve been angry. This was a way of—I think it was the most powerful way—they could get back at someone.”* 

(Coroner 11)

*“I think most of the indigenous suicides do appear to happen around fights and disturbances and are often trivial to us seemingly. But yeah, they’re trying to make a point and it’s sort of revenge in a number of ways.”* 

(Coroner 12)

##### Suicides Are Often in Public

Following on from the previous component of Indigenous suicide, a significant element of a targeted death, is to make that death public. In addition to the accusatorial element of such a public death, there is also the element of dying where other members of the community have died. The little research that explores this in the context of Indigenous Australians, discusses “place” as a site of contagion. Hanssen (2011) identifies that Indigenous suicides often occur at a particular location or place, including the same tree, power pole or water tank [44].

*“The clusters that occur in these communities; you know—the hanging tree and stuff like that, where numerous kids will take their own life at the same spot. You’d have to think that was a fairly graphic manifestation of some shared feeling rather than isolation.”* 

(Coroner 8)

*“In parks. Yeah, and they’re intending to be found there, where everyone can see them.”* 

(Coroner 1)

Hanssen (2011) also maintains that a “public” suicide can occur in or around the home [44]. Such suicides are “public”, due to the overcrowded nature of many Indigenous homes, and the responsibility of providing for an extended family household. Hunter et al. (2006) agree that Indigenous suicide tends to take place in or close to home, visible to members of the family and possibly also to passers by [44].

#### 3.2.2. The Coronial System and Indigenous Families

In contrast to the common model of non-Indigenous suicide discussed previously, Indigenous families often respond to the coronial process in very different ways: they are disengaged from the process; and they experience less stigma when the death is a suicide. Both of these factors mean that they are less likely to publicly resist a finding of suicide and/or agitate within the coronial system to coroners, police or support staff. One consequence of that this may increase the likelihood of coroners reaching a suicide finding for Indigenous deaths.

##### There Is a Disengagement from the Process

For a variety of historical, cultural and legal reasons, Indigenous families do not engage with the coronial process to the same degree as non-Indigenous. These reasons include cultural practices around death that coronial and police inquiries may be deemed to compromise. As a consequence, coroners are not placed under the same degree of pressure by families resistant to a finding of suicide.

*“But also indigenous people are not very engaged with the process at all. They’ll do things, but it’s really hard for them. It’s in another language. I suspect for them it’s just another lot of white people: they come in, they do their whacky thing.”* 

(Coroner 10)

*“I find that Aboriginal families are less likely to agitate about things … I think they are less likely to question it seems to be my experience.”* 

(Coroner 22)

*“The family keeps out of it.”* 

(Coroner 5)

##### There Is Less Stigma for Suicide

Even if Indigenous family involved themselves in the coronial process to the same degree as non-Indigenous, coroners believe they would be unlikely to lobby for non-suicide findings as this carries less social stigma in their community.

*“A significant proportion of our coronial findings in terms of self-harm deaths were related to Indigenous people. So because they don’t have an issue with suicide, you don’t have an issue of finding it if there’s evidence to support it. They do take it for what it is. There’s an ownership. They accept it.”* 

(Coroner 14)

*“I didn’t feel there was a sense of shame.”* 

(Coroner 10)

*“There seems to be less of a cultural issue in relation to kids killing themselves or anyone killing themselves really … the stigma that our society feels, doesn’t exist at all.”* 

(Coroner 12)

#### 3.2.3. Perceptions of Social Disadvantage

Australian coroners appear to have quite conventional ideas about Indigenous life and culture. Many of these are framed in the context of Indigenous Australians who live in remote areas of Australia. For example, a 2008 coronial inquest into a spate of Indigenous suicides concluded the following [45]:

*It was clear that the living conditions for many Aboriginal people in the Kimberley were appallingly bad. The plight of the little children was especially pathetic and for many of these the future appears bleak. Many already suffer from foetal alcohol syndrome and unless major changes occur most will fail to obtain a basic education, most will never be employed and, from a medical perspective, they are likely to suffer poorer health and die younger than other Western Australians. In this context the very high suicide rates for young Kimberley Aboriginal persons were readily explicable*.

One outcome is that suicides can be positioned as more understandable, given their social circumstances. In Australia, numerous studies have demonstrated the presence of a notable geographical difference in age standardised suicide mortality, where suicide rates are generally higher in rural and remote areas and areas with low socio-economic status [46]. This relationship between social and material deprivation and suicide appears heightened for coroners when Indigenous communities are considered, given their known low social and economic status.

*“If you look at the cohort of Aboriginal people who commit suicide, they have to be in that hopeless, helpless situation. They’ve had appalling upbringing, for whatever reason.”* 

(Coroner 27)

*“The way that some people live or are forced to live, is so destructive from the start, and self-destructive with drugs, mental illness, broken homes, that sort of stuff, domestic violence, whatever. It just seems to be the accumulation, really the end point, it was always going to happen.”* 

(Coroner 26)

This has the potential to lead to a circularity of logic. That is, the perception that indigenous people have terrible lives, leads to an understanding/expectation of suicide, which leads to higher Indigenous suicide rates, which increases the perception that indigenous people have terrible lives, etc.

#### 3.2.4. “The Balance of Probabilities”

It is interesting to note that those coroners most comfortable with a 51% appear to be those from states with high indigenous populations—Western Australian, Queensland and Northern Territory. In contrast, coroners from states with far lower Indigenous populations—such as New South Wales and Victoria—require far higher levels of proof in order to reach a finding of suicide.

*“More probable than not … that’s 51 percent. But I think most of us are not really in that realm, we’re really around the kind of 75 percent comfort level, if you want to put a figure on it.”* 

(Coroner 31—VIC)

*“We had a couple of coroners say “If it’s 51%, I’ll find suicide.” (Interviewer). “Yeah, I’m with them.”* 

(Coroner 18—NT)

#### 3.2.5. Capacity

In order to reach a finding of suicide, the individual concerned must be deemed by the coroner to have the capacity to understand the implications of their actions. However, the notion of capacity appears to work differently according to whether or not an individual is Indigenous. The first issue involves alcohol, and the second, age.

##### Alcohol

Alcohol is often used to discount suicide for non-Indigenous Australians by supporting the assertion that they were too drunk to know what they were doing. Certainly in England, intoxication has been used by coroners as grounds not to return a verdict of suicide [47]. For coroners in Australia, acts performed while intoxicated have been found to fall into the grey area of potential accidents due to the impact of alcohol on a capacity to form intent [25]. Coroners interviewed for this research also positioned alcohol as diminishing intent in non-Indigenous suicides.

*“You do more stupid things when you’re alcohol fuelled or drug affected. They’re bad, stupid, risky behaviours with the consequences of death, which I can see was foreseeable given what they’re doing. But whether they’re intentional, whether did they mean to take their own life? No, and I think we should keep it just at that.”* 

(Coroner 30)

However, when coroners were asked to reflect on any patterns associated with Indigenous suicide, they often raised the role of alcohol in the death. Research suggests that recorded Indigenous suicide has a higher history of alcohol and substance use than non-Indigenous suicide, with alcohol implicated in 77% of Indigenous suicide deaths in the Northern Territory ([48], see also [44]). This supports our premise that what coroners determine to be a suicide is what is counted as suicide in the official data.

*“The last Indigenous suicide that I can recall was this lady that I’d forgotten about until it came to mind. She hanged herself in Broome, clearly drunk ...”* 

(Coroner 26)

*“They both seemed to be on a background of a lot of alcohol and what seemed to be a pretty spontaneous thing.”* 

(Coroner 30)

*“… but that’s what you do in certain circumstances when you’re unhappy and someone is against you and you’ve been drinking alcohol and you go do it.”* 

(Coroner 25)

*“If there is a pattern, it’s alcohol, more than anything else. In the sense that they get really charged up.”* 

(Coroner 2)

*“Drunken death would be my definition. It seems to me, the ones that I did in [regional location], they worked up to a position through either drugs or alcohol.”* 

(Coroner 7)

##### Age

A similar issue is raised with respect to age. Coroners are very reluctant to reach a finding of suicide when investigating the deaths of young Australians. It is quite rare for a coroner to find suicide under the age of 15 and the argument proffered is that these individuals are incapable of fully understanding their actions [49].

*“But in my position, it’s the evidence that’s available, and the young child is harder to satisfy yourself that they appreciate the significance of death—they can properly conceive death—and therefore you can’t get the evidence to satisfy yourself that they intended to take their own life.”* 

(Coroner 8)

*“I did have a case involving a 14 year old boy who went in front of a train …. In that case I didn’t make a finding of suicide because it just seemed so random and spur of the moment and unusual for this young person in the context of everything else that I had investigated about his life.”* 

(Coroner 29)

*“A kid at a GPS school, 13 years old, apparently a happy boy, is found hanging in the toilets at school … no broken heart stuff as far as I know, certainly nothing in the stuff that the police found. So, it’s inexplicable really … I just don’t know what he was doing.”* 

(Coroner 1)

This logic is not as readily applied to the death of young Indigenous children. These deaths are found to be suicide, often based upon the premise that these children see so much death in their communities, and in their daily lives, that surely they must grasp its meaning. In fact, despite making up less than three percent of the population, Aboriginal and Torres Strait Islander children and young people represent 28.1 percent of the suicide of deaths by children under 18 [50]. Once again, this lends support to our premise that these statistics are, at least in part, an outcome of the suicide determination of Coroners.

*“I know of Aboriginal youth as young as nine killing themselves. I know of no European of that age. The youngest European I would have thought, off the top of my head, would have been 15 or 16.”* 

(Coroner 18)

*“I do think the fact that other people in your family have repeatedly done that—when I’m talking family, they obviously have a much broader cultural sense of family—then maybe that seems an option. We’ve had little kids do it, like 10 and 12 years old…they’ve seen it and they’ve seen someone hanging off the clothesline before.”* 

(Coroner 25)

*“When you think about how these kids grow up and what they see every day and how they live and what their future is like and what they see as role models and stuff, it’s clearly intentional.”* 

(Coroner 26)

*“I still think many children are in such a devastatingly terrible place that they don’t want to live.”* 

(Coroner 27)

## 4. Conclusions

There are a number of conclusions to be drawn from this research. First, suicide rates are not simply an objective reflection of social truth. They are the product of a complex set of variables that inform and shape the coronial decision-making process. Probably the most significant of these is an ongoing unresolved tension within the role of coroner, a tension between their duty to produce defensible death statistics, and the effects of what has been referred to as “therapeutic jurisprudence”—the belief that coroners should take some responsibility for the emotional well-being of the bereaved families. The most visible effect of this unresolved element of coronial practice is a significant downward pressure on suicide rates.

Second, Indigenous Australians are treated differently within the coronial system. There appears to be far less reticence in reaching a finding of suicide, if the deceased is Indigenous. Therefore, while Indigenous suicide rates are clearly unacceptably high, it may well be that part of the disparity between those figures and non-Indigenous Australians is a greater coronial reluctance to reach a finding of suicide for the non-Indigenous. There is also a circularity in reasoning by coroners, who anticipate a higher suicide rate among Indigenous Australians, and are then part of the mechanism of its production. This finding has already been fed back into the coronial system, and hopefully a greater awareness of the problem will result in an increased sensitivity to all the issues relating to the determination of Indigenous suicide. It may also be the case that this finding has implications for the various processes of suicide determination among other indigenous populations, such as in those in the United States, and in Canada.

Finally, all research into suicide, including that relating to issues of communities in crisis, needs to be aware of some of the shortcomings of the data upon which arguments are often framed, and conclusions drawn. This is not to suggest that the link between social, cultural and political crisis and suicide is not a valid one; it is simply that—given that caution is always required whenever suicide statistics—assessing the strength of that relationship may be more complex than first appears.

## References

[1] Krieg A. (2009). The experience of collective trauma in Australian indigenous communities. Australas. Psychiatry.

[2] Australian Bureau of Statistics (2016). Census of Population and Housing.

[3] Australian Institute of Health and Welfare (2015). The Health and Welfare of Australia’s Aboriginal and Torres Strait Island Peoples: 2015.

[4] Australian Bureau of Statistics (2016). Prisoners in Australia, 2016: Imprisonment Rates.

[5] Australian Institute of Aboriginal and Torres Strait Islander Studies Indigenous Australian Languages. https://aiatsis.gov.au/explore/articles/indigenous-australian-languages.

[6] Australian Bureau of Statistics (2012). Suicides, Australia, 2010: Aboriginal and Torres Strait Islander Suicide Deaths Overview.

[7] Tait G., Carpenter B. (2015). Suicide, Statistics and the Coroner: A comparative study of death investigations. J. Sociol..

[8] Douglas J.D. (1967). The Social Meaning of Suicide.

[9] Platt S., Backett S., Kreitman N. (1988). Social construction or causal ascription: Distinguishing suicide from undetermined deaths. Soc. Psychiatry Psychiatr. Epidemiol..

[10] Timmermans S. (2005). Suicide determination and the professional authority of medical examiners. Am. Sociol. Rev..

[11] Green J. (1992). The medico-legal production of fatal accidents. Sociol. Health Illn..

[12] Community Affairs References Committee (2010). The Hidden Toll: Suicide in Australia.

[13] Australian Bureau of Statistics (2016). Causes of Death, Australia, 2014: Method of Intentional Self-harm.

[14] Ozanne-Smith J., Pearse J., National Coronors Information System (2009). Submission No. 84 to Senate Community Affairs Reference Committee, Inquiry into Suicide in Australia.

[15] Walker S., Chen L., Madden R. (2008). Deaths due to suicide: The effects of certification and coding practices in Australia. Aust. N. Z. J. Public Health.

[16] De Leo D., Dudley M.J., Aebersold C.J., Mendoza J.A., Barnes M.A., Harrison J.E., Ranson D.L. (2010). Achieving standardised reporting of suicide in Australia: Rationale and program for change. Med. J. Aust..

[17] De Leo D. (2015). Can we rely on suicide mortality data?. Crisis.

[18] Studdert D.M., Cordner S.M. (2010). Impact of coronial investigations on manner and cause of death determinations in Australia, 2000–2007. Med. J. Aust..

[19] Palmer B.S., Bennewith O., Simkin S., Cooper J., Hawton K., Kapur N., Gunnell D. (2015). Factors influencing coroners’ verdicts: An analysis of verdicts given in 12 coroners’ districts to researcher-defined suicides in England in 2005. J. Public Health.

[20] Stanistreet D., Taylor S., Jeffrey V., Gabbay M. (2001). Accident or suicide? Predictors of coroners’ decisions in suicide and accident verdicts. Med. Sci. Law.

[21] Pierce J. (2002). Interviewing Australia’s senior judiciary. Aust. J. Political Sci..

[22] Holstein J., Gubrium J. (2012). Active Interviewing. Postmodern Interviewing.

[23] Epstein L. (1990). Interviewing US Supreme Court Justices and interest group attorneys. Judicature.

[24] Heumann M. (1990). Interviewing trial judges. Judicature.

[25] Jowett S., Carpenter B., Tait G. (2018). Determining a suicide under Australian law. UNSWLJ.

[26] Braun V., Clarke V. (2006). Using thematic analysis in psychology. Qual. Res. Psychol..

[27] Carpenter B., Tait G., Stobbs N., Barnes M. (2015). When coroners care too much: Therapeutic jurisprudence and suicide findings. J. Judic. Adm..

[28] Tait G., Carpenter B.J. (2013). Suicide and the therapeutic coroner: Inquests, governance and the grieving family. Int. J. Crime Justice Soc. Democr..

[29] Tait G., Carpenter B., De Leo D., Tatz C. (2015). Problems with the coronial determination of ‘suicide’. Mortality.

[30] Cholbi M. (2007). Self-manslaughter and the forensic classification of self-inflicted death. J. Med. Ethics.

[31] Chapple A., Ziebland S., Hawton K. (2012). A proper fitting explanation? Suicide bereavement and perceptions of the coroners verdicts. Crisis.

[32] White J., Olson K., Young R., Schultz I. (2015). Qualitative evidence in suicide. Handbook of Qualitative Health Research for Evidence-Based Practice.

[33] Cavanagh J.T., Carson A.J., Sharpe M., Lawrie S.M. (2003). Psychological autopsy studies of suicide: A systematic review. Psychol. Med..

[34] Tarrier N., Gooding P., Pratt D., Kelly J., Awenat Y., Maxwell J. (2013). Cognitive Behavioural Prevention of Suicide in Psychosis: A Treatment Manual.

[35] Carpenter B.J., Tait G., Adkins G., Barnes M., Naylor C., Begum N. (2011). Communicating with the coroner: How religion, culture, and family concerns may influence autopsy decision making. Death Stud..

[36] Tait G., Carpenter B., Quadrelli C., Barnes M. (2016). Decision-making in a death investigation: Emotions, families and the coroner. J. Law Med..

[37] Freckleton I. (2008). Therapeutic jurisprudence misunderstood and misrepresented: The price and risks of influence. Thomas Jefferson Law Rev..

[38] Tatz C. (2005). Aboriginal Suicide Is Different.

[39] Cuneen C. (2006). Aboriginal deaths in custody: A continuing systematic abuse. Soc. Justice.

[40] Carpenter B., Tait G., Quadrelli C., Drayton J. (2016). Scrutinising the other: Incapacity, suspicion and manipulation in a death investigation. J. Intercult. Stud..

[41] Carpenter B.J., Tait G.W. (2009). Health, death and Indigenous Australians in the coronial system. Aust. Aborig. Stud..

[42] Tighe J., McKay K., Maple M. (2015). “I’m going to kill myself if you don’t …”: Contextual aspects of suicide in Australian Aboriginal communities. Int. J. Cult. Ment. Health.

[43] Hunter E., Milroy H. (2006). Aboriginal and Torres Strait Islander suicide in content. Arch. Suicide Res..

[44] Hanssen L. (2011). Suicide (Echo) Clusters. Aborig. Isl. Health Work. J..

[45] Coroners Court of Western Australia (2008). Inquest into the Deaths of—Edward John Riley, Rachael Henry, Chad Atkins, Teddy Beharral, Maitland Brown, Jonathon Dick, Lloyd Dawson, Benjie Dickens, Ivan Barry Gepp, Owen Gordon Jonathan Hale, Ernest James Laurel, Joshua Middleton, William Robert Miller, Gordon Oscar, Celeste Antoinette Shaw, Shawn Surprise, Davina Kaye Edwards, Nathalia Maree Cox, Desley Sampi, Llewellyn Sampi, Troy James O’sullivan, Zedrick Yamera.

[46] Law C.K., Snider A.M., De Leo D. (2014). The influence of deprivation on suicide mortality in urban and rural Queensland: An ecological analysis. Soc. Psychiatry Psychiatr. Epidemiol..

[47] Linsley K.R., Schapira K., Kelly T.P. (2001). Open verdict v suicide: Importance to research. Br. J. Psychiatry.

[48] De Leo D., Sveticic J., Milner A., McKay K. (2011). Suicide in Indigenous Populations of Queensland.

[49] Martin G. (2015). Editorial on child suicide. Adv. Ment. Health.

[50] Mitchell M., Gooda M. (2015). Self harm and help seeking among Aboriginal and Torres Strait children and young people. Indig. Law Bull..

